# Complex genetic architecture underlies human hand and foot evolution

**DOI:** 10.21203/rs.3.rs-7124496/v1

**Published:** 2025-08-18

**Authors:** Alexander Okamoto, Gayani Senevirathne, Pushpanathan Muthuirulan, Campbell Rolian, Ian Glass, Birth Defects Research Laboratory, Terence Capellini

**Affiliations:** Harvard University; Harvard University; Harvard University; McGill University; 11. Division of Genetic Medicine, Department of Pediatrics, University of Washington, Seattle, Washington; University of Washington; Harvard University

## Abstract

The transition to bipedal locomotion is a key event in human evolution, involving substantial changes to the skeleton, including the bones of the hands and feet (autopods). Hominins evolved a more muscular and opposable thumb while the other fingers are relatively shorter, enhancing manipulative capacity. The feet evolved robust first toes and short lateral toes to meet the challenges of bipedal walking and running. While adaptations in the hand and foot have often been considered separately, the fore- and hind limbs of primates are morphologically integrated, serially homologous structures, raising the possibility that natural selection on either autopod may have driven corresponding changes in the other. To explore the genetic architecture underlying human autopod evolution, we used functional genomics methods to identify regulatory elements and gene expression patterns in the developing phalanges and metacarpals of the human hand and foot. We find that gene expression and regulation differ along the proximal-distal axis and between timepoints but not between limb types or individual digits. We show that thousands of human-specific genomic features fall within autopod regulatory elements, some accessible in multiple tissues, others with tissue-specific accessibility. Our results highlight the complex genomic basis of human autopod evolution.

The functional divergence of the hand and foot for prehension and locomotion, respectively, has long been recognized as a critical event in human evolution^1^. In comparison to other apes, the human hand and foot have features in skeletal anatomy as well as the related musculature and innervation which are believed to have evolved adaptively in the context of their respective functional specializations^2–5^. Skeletally, the human hand evolved a large pollex (i.e., thumb) and a reduced length of the other digits, which improved manipulative capacity, a key factor in successful tool use for food acquisition and processing, and defense^6–10^. The human foot evolved a robust, adducted hallux (i.e., big toe) and short digits, creating a shorter, hinge-like forefoot with a stiff midfoot that improves performance in both walking and running^11–15^. These derived features contribute to the unique status of humans as the only extant primates that are obligate bipeds.

Due to their differences in structure and function, the evolutionary histories of the human hand and foot have largely been considered separately^3,6–14,16–18^. However, data from both developmental genetics and hominoid anatomical studies provide evidence that the evolution of the hand and the foot should be considered together: hands and feet (hereafter collectively referred to as autopods) are serially homologous structures, share much of their genetic architecture^19^, and have been shown to covary strongly in size and shape^20,21^. Furthermore, when the anatomical changes between human and chimpanzee autopod skeletons are compared for both limb types, it becomes clear that some changes are remarkably similar – e.g., a larger, more robust first digit compared to the other digits, shorter phalanges compared to metapodials, loss of phalangeal curvature, and an overall size reduction (Fig. 1a–c). This raises the possibility that phenotypic changes in either the hand or foot might in part be explained by adaptive evolution in the other due to the potentially constraining effects of strong covariation between homologous limb elements of humans and other apes^20–22^. Simulations even suggest that selection for a modern human-like foot from a chimp-like ancestor is sufficient to produce a human-like hand purely as a byproduct due to anatomical integration of the autopods^23^.

Despite the importance of the hand and foot in human evolution, very little is known about the genetic mechanisms patterning human-specific features of the hand and foot skeleton. Many of these features arise early in human development when the limb skeleton is first patterned in cartilage templates (Fig. 1d). While some studies have investigated the developmental genetics of human limbs during or slightly before this developmental window^24,25^, none have generated matched datasets for the hand and foot, nor for individual skeletal elements of each autopod. Therefore, to shed new light on the evolutionary history of the human hand and foot skeleton, we generated data on the gene expression and regulation of each metapodial and the pooled phalanges of each digit in both the hand and foot at two stages during this developmental window. Additionally, we performed corresponding experiments in stage-matched E15.5 mouse embryos on digits I, III, and V to explore evolutionary conservation of identified genes and regulatory elements. Using these datasets, we investigated the signature of natural selection on genomic regions patterning distinct elements of the human hand and foot skeleton. We tested (1) the prediction that there would be substantial overlap in gene expression and the genomic regulatory landscape between the hand and foot tissues (the genetic underpinnings of covariance and coevolution), and (2) that the foot would show stronger signatures of selection than the hand, in line with previous hypotheses and the fact the remodeling of the foot in the context of bipedalism was more extensive and predates the appearance of lithic technology^6^.

## Proportional changes in the autopod skeleton

**Figure 1:**
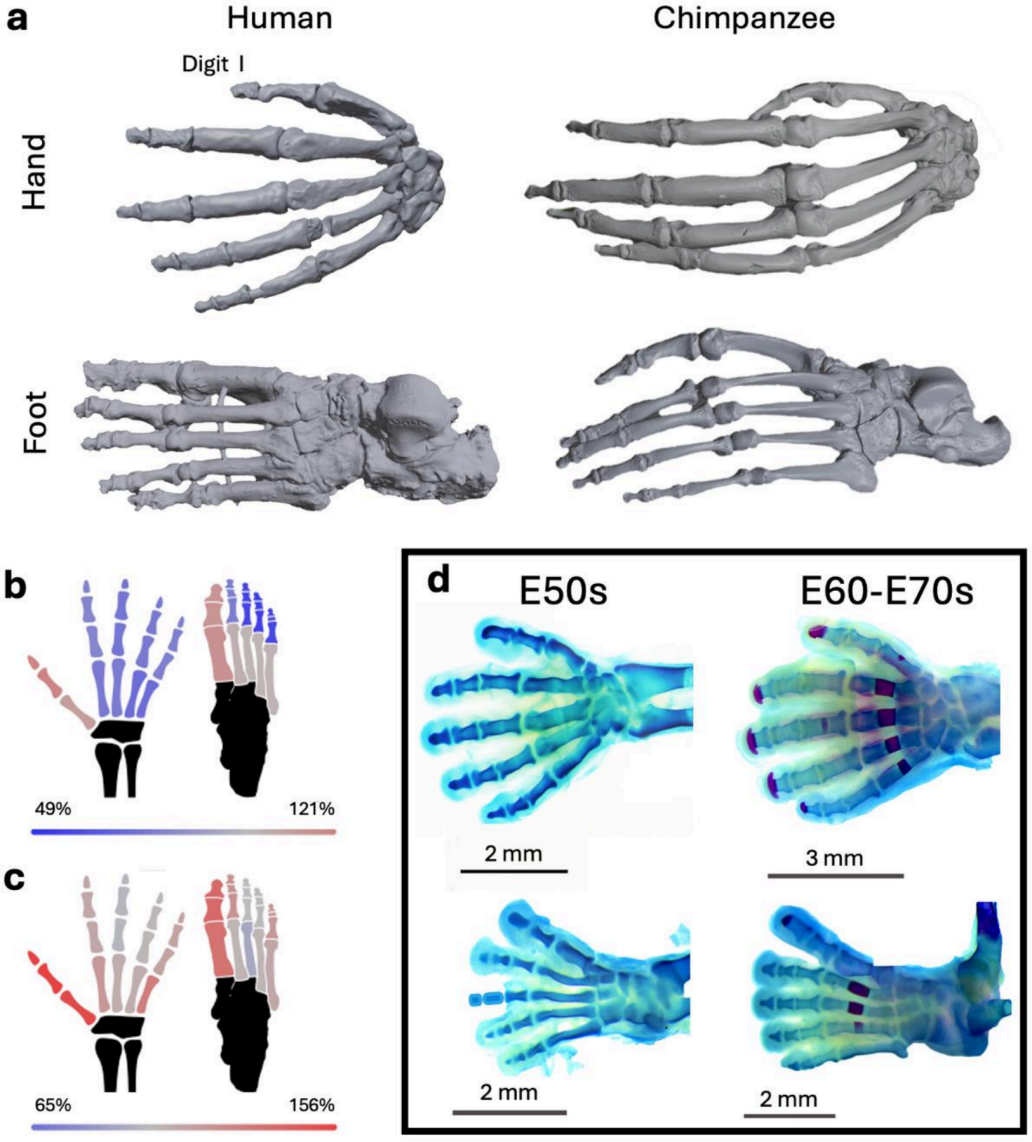
Anatomy of the human and chimpanzee autopod skeleton **a**, morphology of the adult hand (top) and foot (bottom) skeleton in human (left) and chimpanzee (right). **b**, average percent change in length and **c**, width between adult human and chimpanzee autopod skeletal elements. **d**, alizarin red/alcian blue bone/cartilage staining of developmental human autopod skeletons at gestational day E54 and E67. The intermediate and distal phalanges of digit III in the E50s sample were lost during sample preparation and have been drawn in. Metatarsal I in the E60–70s sample is broken and has been digitally corrected. Note that the phalanges are splayed due to the removal of soft tissue. Photos courtesy of Mariel Young.

To quantitatively assess anatomical differences in proportion across the autopod skeleton, we first estimated the average percent difference in the length and width of each element in a large dataset of adult human, chimpanzee, and gorilla skeletons (See Methods) (Supplementary Table 1). As expected, the greatest amount of difference was observed in the more robust first digit in humans when compared with chimps (Fig. 1b–c). The other digits were generally shorter – especially the foot phalanges – with the exception of the metacarpals, which were similar in length between the two species (Figs. 1b–c). The same pattern is observed when humans are compared to gorillas (Extended Data Fig. 1).

## Patterns of gene expression and regulation across the autopod

**Figure 2:**
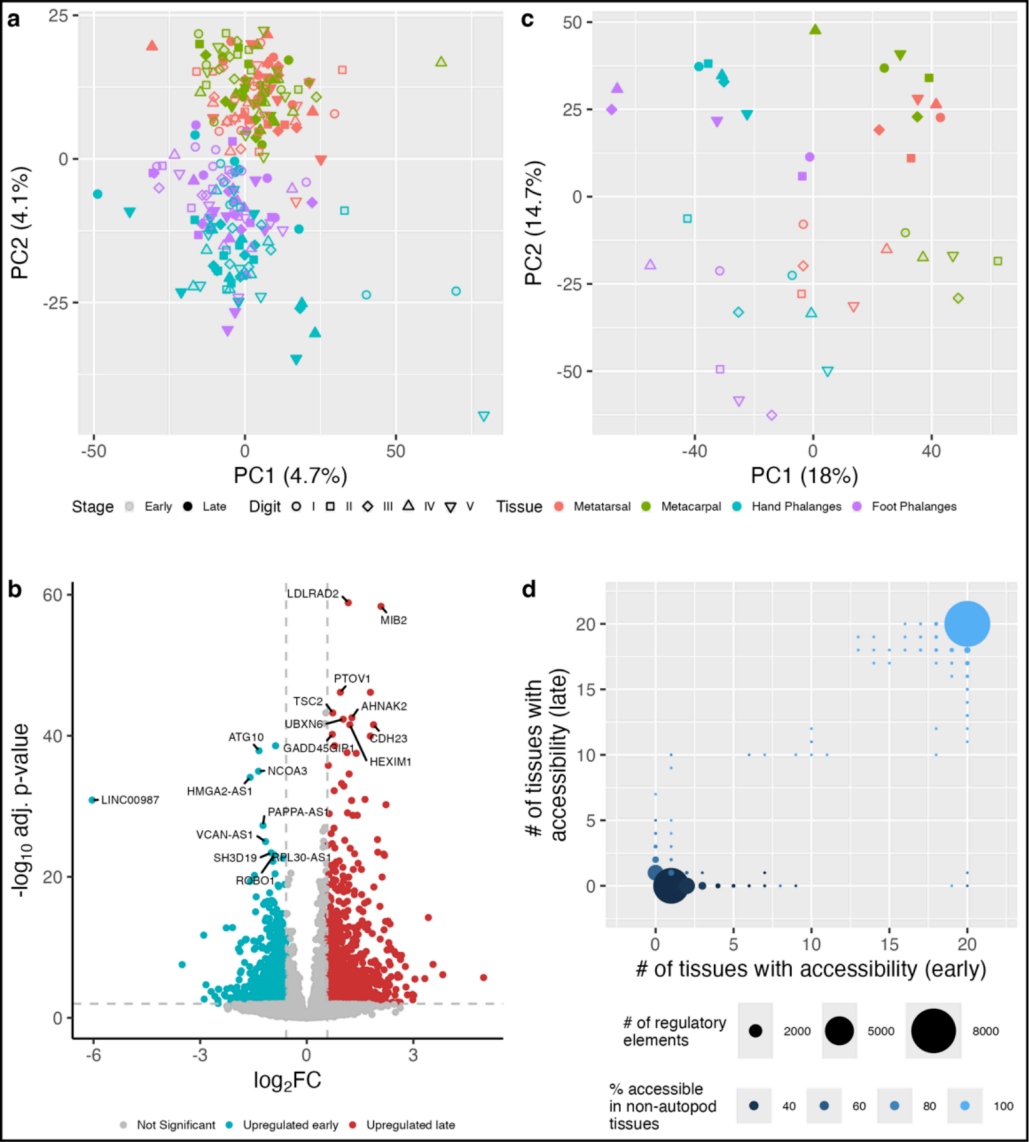
Gene expression and regulation in the human autopod **a**, Principal component analysis (PCA) of normalized human gene count data, corrected for biological replicate. **b**, Volcano plot showing the differences in gene expression between early and late samples. Timepoint differences are not seen in **a**, due to correction for biological replicate in the Deseq2 model. **c**, PCA of normalized accessibility data corrected for variation between biological replicates. The top 10 most significantly differentially expressed genes early and late are labeled. **d**, Accessibility sharing of regulatory elements across timepoints. Dot sizes show the summation of all tissue combinations with the same total number of tissues with accessibility at each timepoint. Only tissue combinations with at least 5 regulatory elements were included in this calculation. See Methods for the full list of non-autopod skeletal tissues considered in this analysis.

The identified proportional differences most likely reflect modifications to autopod development in each species, especially at the cartilage level. This is because the rudiments of each digital element are comprised of chondrocytes that organize to form growth plates governing longitudinal and transverse growth during ontogeny. We examined human foot and hand development and identified two gestational timepoints (E54 and E67) spanning a window where morphogenesis was occurring rapidly (Fig. 1d, Supplementary Note). For each digital ray of the human hand and foot, we generated matched transcriptomic (RNA-seq) and epigenomic (ATAC-seq) datasets for the phalangeal and metapodial regions separately (Supplementary Tables 2–3, Supplementary Results S1–3). For comparative purposes, we performed a similar approach on stage-matched mouse E15.5 forelimb and hind limb elements, focusing on digits I, III, and V (Extended Data Fig. 2, Tables S3–5, Supplementary Results S4–5). Analysis of our human RNA-seq dataset identified 1,263 differentially expressed genes (DEGs) between biologically relevant sets of autopod tissues (See Methods; Supplementary Table 2). These comparisons included adjacent tissues (along either the anterior-posterior or proximal-distal axes), homologous tissues between the hand and foot, and the same tissue across timepoints, as well as gene expression gradients across the autopod. Many DEGs separated the metapodials and phalanges but not individual digits (Fig. 2a; Extended Data Fig. 3). As predicted based on the patterns of covariance of coevolution, gene expression was overwhelmingly similar between the hand and foot, consistent with the expression patterns we observed in mouse (Extended Data Fig. 2)^26,27^. We also identified 3,031 DEGs between the two timepoints, with 1,453 genes upregulated early at E54 and 1,578 genes upregulated late at E67 (Fig. 2b). The later developmental time point was enriched for gene ontology (GO) terms related to “ossification,” which initiates around this stage.

Using our human ATAC-seq data, we identified 60,150 regulatory elements accessible in the chondrocytes of at least one autopod skeletal element (Supplementary Table 3). Consistent with the RNA-seq data, principal component analysis (PCA) separated samples between the metapodials and phalanges and between timepoints but not by limb type (Fig. 2c). Indeed, no elements uniquely defined all hand or all foot tissues. Overall, regulatory elements are often accessible in many autopod tissues or tissue and timepoint specific (Fig. 2d). Using our previously collected stage-matched ATAC-seq datasets from human long bones, girdles, and the axial column^28–30^, we found that many of the identified regulatory elements show accessibility in at least one other developing skeletal tissue, albeit 12,488 regulatory elements have not been previously identified and appear unique to the autopod (Fig. 2d). To help elucidate broader regulatory patterns in functionally conserved sequences in other primates, we asked how many of the human autopod elements overlapped elements present in mouse, since elements conserved between human and mouse are likely present in other primates. While the number of individual rays examined in mouse was 3 versus 5 in humans, we found that 20,491 regulatory elements were accessible in both human and mouse (34.0% of all regulatory elements identified in human), only 2,654 of which were autopod-specific (21.3% of all human autopod-specific elements) (Extended Data Fig. 4).

## Genomic evolution in autopod regulatory elements

**Figure 3:**
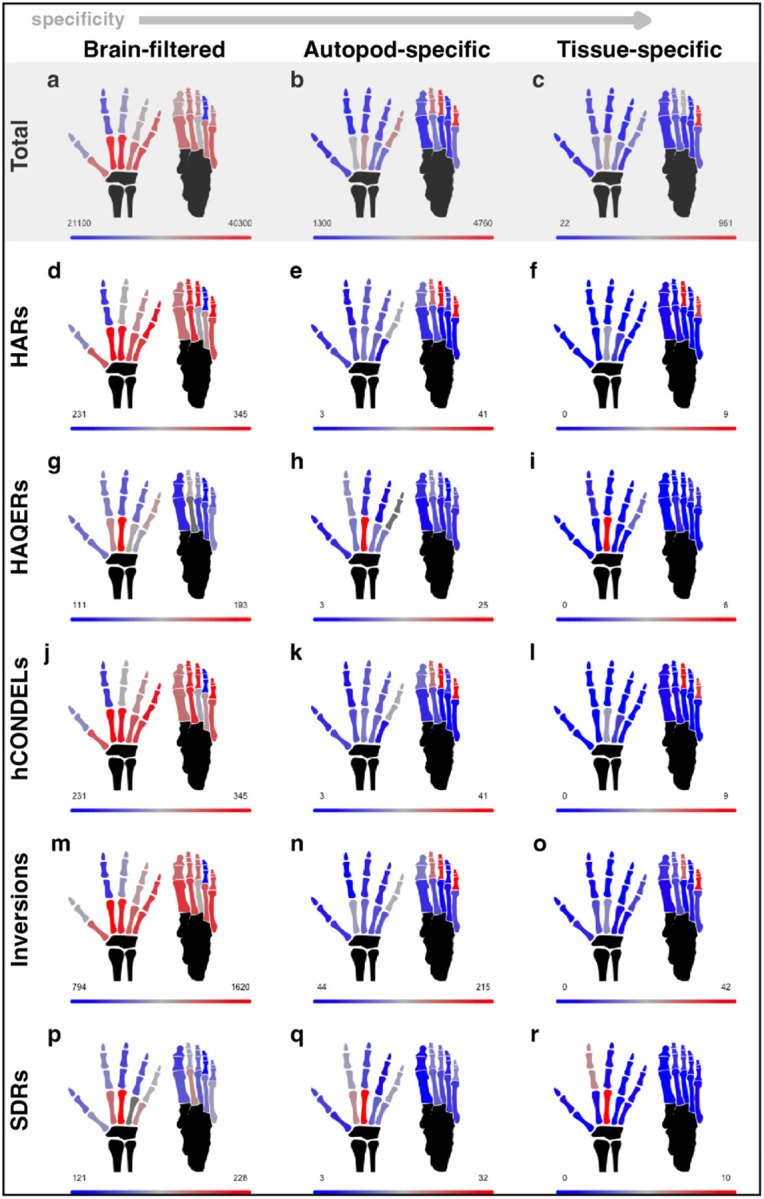
Evolutionary signals overlapping autopod regulatory elements **a**, total number of regulatory element accessible in each tissue after brain-filtering. **b**, number of regulatory elements in each tissue that are accessible only in autopod tissues, and **c**, number of regulatory elements that are accessible in only a single tissue at one or both timepoints. Number of overlaps for the brain-filtered (**d, g, j, m, p**), autopod specific (**e, h, k, n, q**), or tissue-specific (**f, i, l, o, r**) regulatory element sets with HARs (**d-f**), HAQERs (**g-i**), hCONDELs (**j-l**), major human inversions (**m-o**), or human SDRs (**p-r**).

Using our dataset, we sought to investigate the genomic basis of human-specific features of the autopod skeleton. To this end, we overlapped our autopod regulatory element sets with genomic regions marked by signatures of human-specific changes, including human accelerated regions (HARs)^31–38^, human conserved element deletions (hCONDELs)^39,40^, human ancestor quickly evolved regions (HAQERs), large human-specific inversions, and structurally divergent regions between humans and other apes (SDRs)^41^ (Table 1, Extended Data Fig. 5–6). As structural changes can impact neighboring genomic loci by rearranging the local chromatin environment, overlaps were also determined for regions flanking each inversion and SDRs (Extended Data Fig. 7). We identified overlaps for these different types of genomic regions for elements with accessibility in multiple skeletal or autopod tissues, as well as those that are tissue and timepoint-specific (Fig. 3). Overall, 95% of the genes expressed in at least one autopod tissue fell within 500kb of one or more of these genomic regions. While all tissues exhibited accessibility for at least a few regulatory elements overlapping each type of genomic feature, this was not true for tissue-specific elements (Fig. 3f,i,j,o,r). Tissues with high levels of morphological change were not distinctly enriched for evolutionary signals, with relatively few overlaps detected in digit I but large numbers of overlaps seen in the foot phalanges of digits III and V (Fig. 3d–f,j–o). This analysis revealed that thousands of genomic loci with accessibility in the developing human autopod skeleton harbor evolutionary sequence alterations.

**Figure 4:**
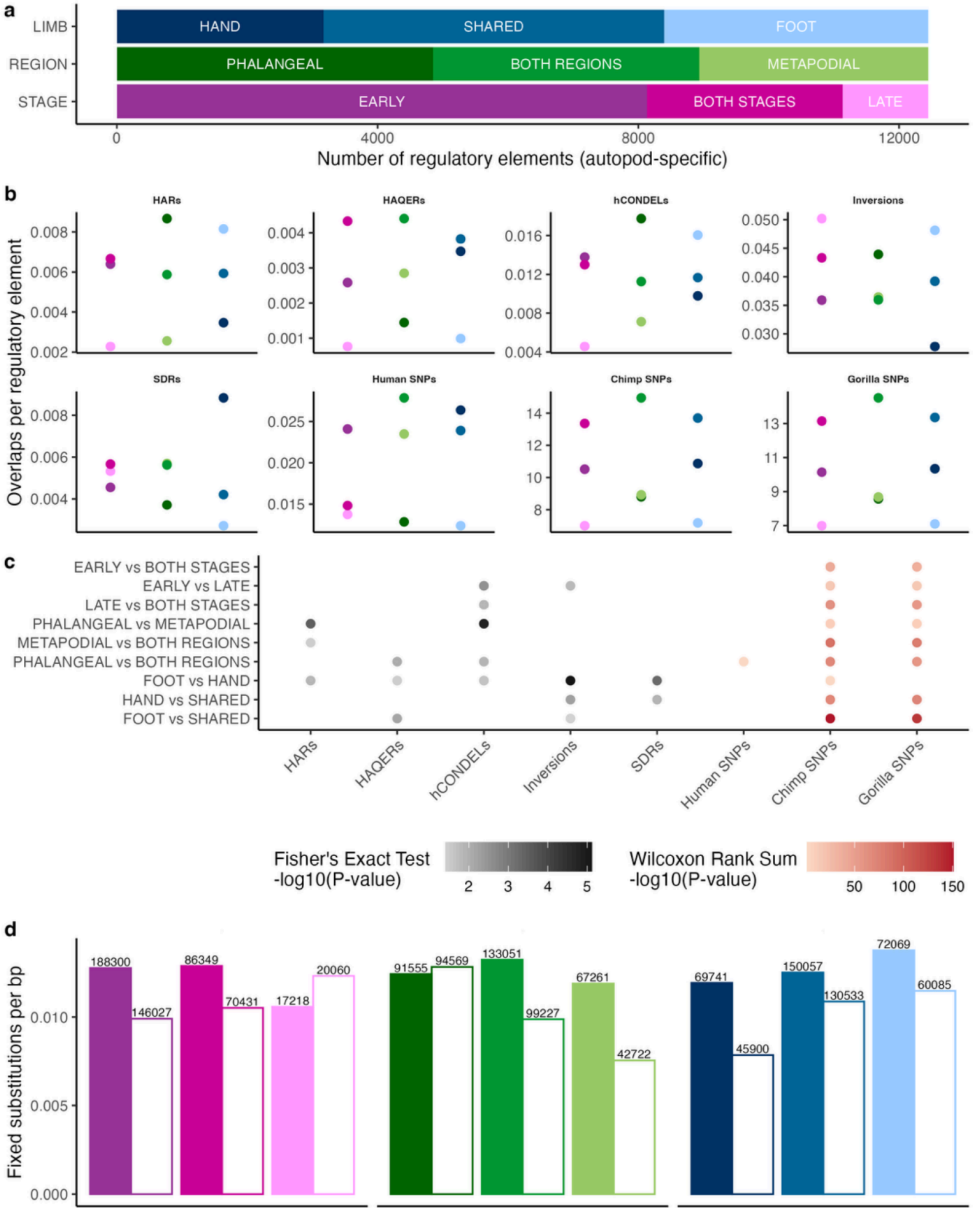
Partitioning of evolutionary signals across the autopods **a**, division of regulatory elements accessible only in autopod tissues by the specificity of accessibility in limb type, proximal-distal region, or developmental stage. **b**, enrichment of each regulatory element set for HARs, HAQERs, hCONDELs, human-specific inversions, human SDRs, as well as common variants (MAF > 0.05) in human, chimpanzee, and gorilla per base pair of sequence. **c**, statistical significance of enrichment differences between set pairs. **d**, number of fixed substitutions along the human (colored bars) and chimpanzee (white bars) lineages per base pair for each set. Total number of fixed substitutions are listed above each bar. Color scheme in **b,d**, is the same as in **a**.

To link these genomic changes to anatomical subdivisions of the autopod, we sought to partition our regulatory elements and overlaps according to their accessibility patterns. Based on the differences in gene expression and regulation between the phalanges and metapodials and between developmental stages, as well as the anatomical differences between the hand and foot, we explored the relative strength of the evolutionary signals along each of these axes (Fig. 4a–c). Given that evolutionary forces, such as natural selection, shape the patterns of genetic variation present within a species, we also compared the patterns of human, chimpanzee, and gorilla intraspecific variation within each regulatory set (See Methods). Enrichments within each comparison differed depending on the genomic feature and the specificity of the regulatory element set considered (i.e., all brain-filtered elements, autopod-specific elements, or autopod-specific, human-mouse conserved) (Fig. 4, Extended Data Figs. 8–9). Within the autopod-specific sets, the most significant differences in enrichments were identified for the hand versus foot comparison (Fig. 4c).

For the autopod-specific elements, we sought to explore biological differences between these sets. To test whether different sets regulated the same or distinct target genes, we counted the number of regulatory elements from each set that fell within 500kb of genes expressed in at least one autopod tissue (Additional Data Fig. 10). As expected from the largely similar pattern of gene expression observed in all tissues, we found that the number of nearby regulatory elements from the shared sets were largely correlated with those of the more restricted ones. For the more specific sets, the numbers of hand and foot elements were not correlated (Pearson’s Correlation, R = 0.02, P = 0.365), while the numbers of phalangeal and metapodial elements were positively correlated (R = 0.11, P < 0.001) and the early and late elements negatively correlated, although given the strength of the correlation, this is unlikely to be biologically meaningful (R = −0.075, P < 0.001). Overall, many genes are near regulatory elements from multiple categories, consistent with complex regulatory control of gene expression. Accordingly, gene ontology enrichments for each set differed slightly but all included terms related to cartilage or bone development (Supplementary Table 3). Finally, we analyzed transcription factor (TF) binding profiles and found that the sets were differentially enriched for hundreds of TF motifs, suggesting that regulatory elements in each set preferentially interact with distinct sets of TFs (Supplementary Table 3).

While hotspots of human-specific sequence changes are frequently prioritized, single nucleotide changes may also have substantial phenotypic consequences. Accordingly, we sought to estimate the total number of nucleotide changes that may have contributed to human autopod evolution. Briefly, we took our regulatory elements, removed polymorphic variant positions and misaligned regions, and counted the number of positions where the reference nucleotide in the human genome differed from that present in both the chimp and gorilla genomes (see Methods). While this approach ignores structurally divergent and misaligned regions, it gives a rough minimum estimate of the number of fixed substitutions that may contribute to human-specific differences. Using this approach, we identified 2,461,335 human-specific fixed substitutions that are accessible in the developing autopod skeleton, 291,867 of which are autopod-specific. Since humans and chimpanzees share the same last common ancestor to gorilla and therefore have evolved independently for the same length of time (i.e., the branch lengths are equal), we repeated this process using the chimpanzee branch. We identified 2,012,269 chimp-specific fixed substitutions that are accessible in the developing autopod skeleton, 236,518 of which are autopod-specific. Many of these changes likely resulted from neutral evolutionary processes and have minimal or no phenotypic effects, however, comparison of PhyloP scores between the human and chimp sets found a significant shift towards more positive scores in human (human mean 0.23, chimp mean 0.01, *P* < 2.2 × 10^− 16^, two-sided Wilcoxon rank-sum), suggesting that human fixed changes alter positions with deeper evolutionary constraint. This holds true for the autopod-specific set as well (human mean 0.24, chimp mean 0.05, *P* < 2.2 × 10^− 16^). Overall, this analysis reveals that ~ 450,000 more fixed substitutions in autopod regulatory elements occurred on the human branch than the chimpanzee branch, with ~ 55,000 accessible only in the autopod. We partitioned these fixed substitutions along the same anatomical axes discussed above, finding that except for the elements unique to phalangeal and or late tissues, all sets showed more fixed substitutions along the human branch (Fig. 4d).

## Discussion

The evolution of bipedalism is a critical event in hominin evolution and involved substantial remodeling of the postcranial skeleton. To further our understanding of the genetic mechanisms underlying these skeletal changes, we used functional genomics methods to investigate gene expression and regulation during development of the distal autopod skeleton in human. We identified thousands of regulatory elements and genes involved in the developing human hand and foot skeleton. These data revealed substantial differences in gene expression and regulation between the phalanges and metapodials and between the two timepoints but not between digits or limb types. While some of the identified regulatory elements are highly spatially and temporally restricted, most are accessible in multiple skeletal tissues, both within the autopod and across the postcranial skeleton. These results provide a genetic basis for the observed patterns of covariation across the autopod skeleton.

We found that both multi-tissue and tissue-specific regulatory elements overlap thousands of genomic features that differ between humans and chimpanzees and could potentially underlie the evolution of human-specific autopod traits; these genomic features include human-specific nucleotide changes, losses or gains of sequence, as well as structural variation. Elements broadly accessible in autopod tissues provide a genetic basis for models of human limb evolution based on strong morphological integration (i.e. elevated phenotypic covariance) between homologous elements of the fore- and hind limbs^20,21^. While autopod covariation is relatively weaker in comparison to quadrupedal monkeys, homologous elements of the hands and feet of humans and other apes still covary substantially, suggesting the evolution in one limb could be driven at least in part by selection on the other^20,21,42^. Indeed, simulations suggest that selection for human-like foot proportions in a chimpanzee-like ancestor is sufficient to drive the evolution of the human hand, while the converse is not true^23^. The data presented in this study provide some support for this model. While we found many human-specific genomic features in regions accessible in multiple tissues, consistent with phenotypic covariation between elements, support for stronger selection on the foot depends on the evolutionary signal considered. In regulatory elements accessible only in autopod tissues, we found significantly more HARs, hCONDELs, and inversions but fewer SDRs within regulatory elements patterning the foot than the hand (Fig. 4b,c). We also found lower levels of intraspecific variation in the foot than the hand in human, chimpanzee, and gorilla, consistent with stronger stabilizing selection on the foot in each lineage. Finally, while there are overall more fixed substitutions found in foot elements than hand elements, both absolutely and per base pair, there is a greater excess of human compared to chimpanzee fixed substitutions in the hand (Fig. 4d). These patterns generally hold when considering the sets of all elements, autopod-specific elements, or human-mouse conserved autopod elements, but there are some differences, such as significantly more HAQERs overlapping hand than foot regulatory elements in the total set of brain-filtered elements. An important caveat of our approach is that as we rely on accessibility data from extant human samples, these patterns may not reflect the ancestral state.

Altogether, we estimated that there are almost 2.5 million fixed nucleotide substitutions in the human genome compared to chimpanzee and gorilla that are accessible to transcriptional machinery in the developing autopod skeleton. This is ~ 450,000 more fixed nucleotides than are fixed in chimpanzee. Together with the fact that the human fixed nucleotides disrupt more evolutionarily conserved positions, this suggests a potentially greater extent of anatomical evolution along the human branch. ~55,000 of these fixed substitutions are uniquely accessible in autopod tissues, suggesting that autopod evolution likely has a complex underlying genetic architecture, even without considering integration with other elements of the skeleton. To the best of our knowledge, this is the first attempt to assign a minimum number of genome-wide fixed substitutions to a given set of human-specific phenotypic changes and highlights the extent of genomic changes that have potentially contributed to the evolution of the human autopod skeleton.

Linking these genomic changes to observed evolutionary changes in anatomy remains a major challenge^43,44^. While regulatory element sets that are uniquely accessible in tissues with a particular trait might offer the most tempting candidate(s) for identifying the genomic changes underlying evolution in that trait, this approach to connecting genotype to phenotype excludes large numbers of regulatory elements that are biologically plausible by implicitly assuming that traits evolve independently^22^. In fact, our data demonstrate that many disparate skeletal elements share an underlying genetic architecture. These shared regulatory elements could be targeted by selection on a single skeletal trait so long as the off-target effects were minimal or could reflect more complex patterns of coevolution across skeletal elements. When we partition our data between the hands and the feet, between timepoints, or between phalanges and metapodials, in each case we find many shared regulatory elements that overlap human-specific changes just within the autopod-specific set. Many more regulatory elements are potentially evolutionarily relevant when elements shared with other postcranial bones are considered and even more would be identified if we removed the brain filtering step of our data processing pipeline. While substantially more challenging to interpret, elements accessible in multiple tissues are an important part of the genomic architecture patterning these skeletal elements.

Of course, many of the human-specific sequence features highlighted in this study likely have no impact on gene expression. While estimating the number and magnitude of effect on gene expression for all these sequences features is beyond the capacities of current technologies, limited experimental research has shown that for regulatory HARs for which both the human and chimp sequence have been tested in transgenic animals, a third resulted in differential enhancer activity^45^. Massively parallel reporter assay testing of hCONDELs found that 8% exhibited differential enhancer activity between human and chimps in at least one cell line^40^. If HARs and hCONDELs show similar levels of activity in the developing skeleton, that would suggest that 158 HARs and 71 hCONDELs have altered regulatory activity in the developing human autopod skeleton (considering elements with accessibility in both autopod and non-autopod tissues, 25 HARs and 13 hCONDELs with accessible only in the autopod). Even if HARs and hCONDELs are substantially more predictive of human-specific gene regulatory evolution than the other sequence features considered, an assumption that has not been rigorously tested, this still suggests that the genomic basis of autopod evolution involved hundreds of genomic loci and should be considered highly polygenic. Under such a model, each individual genomic change is expected to have only a small phenotypic contribution, potentially complicating attempts to validate their effects in humanized mouse models^46^.

To fully understand the genetic underpinning of human autopod evolution, future studies are necessary to generate comparable data on the bones of the wrist, midfoot, and hindfoot. These proximal autopod bones have also undergone substantial evolution in the human lineage^5,14,47,48^ and are undoubtedly directly relevant to the evolution of the metapodials and structure of the autopod in general. Similarly, future studies should investigate the genomic changes underlying evolution in the soft tissues of the autopod. As this study was based on bulk chondrocyte tissue dissections, future research would also benefit from techniques with greater spatial resolution, such as spatial multi-omics, to unravel the genetics basis of these human-specific features with greater anatomical precision (e.g., Senevirathne et al., under review). Such methods could investigate human-specific changes in shape related to load bearing and the articulation of bones with one another. Furthermore, these methods could explore the impacts of extrinsic signaling and mechanical impacts from neighboring tissues, such as mesenchyme and muscle, on autopod skeletal development^2^. Given the intricate functional, developmental, and evolutionary relationships between all parts of the autopod, a holistic approach that integrates datasets from multiple tissue types will ultimately be necessary to understand the evolution of human-specific features. While only part of the story, this study provides novel insights into the evolutionary history of human bipedalism by highlighting the complex genetic architecture underlying human autopod evolution in the phalanges and metapodials.

## Materials and Methods

### Estimated human-chimpanzee anatomical change

Measurements were taken of the length and head width of metapodials, proximal, and middle phalanges of the hand and foot in adult human (*n* = 48), chimpanzee (*n* = 44), and gorilla (*n* = 57) skeletons (Appendix, Table 5). Chimpanzee and gorilla measurements come from wild-shot populations housed in the Powell-Cotton Museum and Hamman Todd Osteological Collection at the Cleveland Museum of Natural History, while human measurements are from the Hamman Todd Collection only. See ref^20^ for details. The proximal and middle phalanges measurements were combined for each digit. The human-chimpanzee percent change for the length and width of each element was calculated as the mean human measurement/the chimpanzee measurement * 100. These calculations were repeated using the gorilla measurements in place of chimpanzee and to compare chimpanzee to gorilla.

### Human developmental sample collection.

Human developmental samples were collected from first-trimester termination through the Birth Defects Research Laboratory (BDRL) at the University of Washington in full compliance with the ethical guidelines of the National Institutes of Health (NIH) and with the approval of the University of Washington Institutional Review Boards (IRB) for the collection and distribution of human tissues for research and Harvard University for the receipt and use of such materials. The BDRL obtained written consent from all tissue donors. Harvard University IRB determined that these samples constitute Non-Human Subjects Determination Status (Capellini: IRB16–1504). The fresh human samples were briefly washed in Hanks’ balanced salt solution and shipped at 4°C. Upon arrival, the samples were immediately dissected under a light dissection microscope and directly subjected to RNA-seq or ATAC-seq protocols described below, following approved Harvard University IRB (IRB16–1504) and Committee on Microbiological Safety (COMS) (18–103) protocols. The BDRL performs polymerase chain reaction (PCR) using SRY and *Amelogenin* primers to determine the biological sex of each sample.

### Human ATAC-seq data collection and processing.

Developmental samples were microdissected under a light microscope in 5% fetal bovine serum (FBS) in Dulbecco’s Modified Eagle Medium (DMEM) on ice. The pooled phalanges (proximal and distal for digit I; proximal, intermediate, and distal for digits II-V) and metapodials were collected separately for all digits in both the fore- and hind limbs. Samples were collected at two timepoints: an early stage (E53 to E59, *n* ≥3) and a later stage (E67 to E74, *n* ≥ 3) to capture the window of time when the autopod skeleton is prepatterned in cartilage and is just beginning to ossify and to account for heterogeneity in the timing of forelimb or hind limb as compared to overall limb development^49,50^.

The tissue in each sample was then digested using 0.5% collagenase II in 5% FBS/DMEM in a 37°C water bath for one hour. Every 30 minutes, the samples were spun down and gently pipetted to break up large clumps of cells. Next, the samples were incubated at 37°C for an additional hour in a shaking incubator. Following these incubation steps, the sample were immediately placed on ice and then filtered through a 70 μm nylon cell strainer into a 50 mL conical tube by gently pressing any residual tissue through the filter followed by rinsing with 5% FBS/DMEM. Sample were centrifuged for 5 minutes at 500*g* at 4°C and most of the media aspirated. The cells were then resuspended and transferred to a 1.5 mL tube before another 5-minute centrifuge at 500*g* at 4°C. At this stage, live and dead cells were counted to ensure that all tissues had ~ 50,000 cells and cell death rates below 10%. 50,000 cells per technical replicated were resuspended in 1x PBS and then lysed using the ATAC-seq lysis buffer and centrifuged for 10 minutes at 4°C^51^. The supernatant was discarded and 50 μl transposition mix containing 2.5 μL transposase was added before the samples were incubated at 37°C for 30 minutes. Lastly, the Zymo DNA Clean and Concentrator kit was used, and the resulting purified DNA was eluted in 13 μl of water heated to 60–70°C. Samples were stored at −20°C prior to PCR amplification and barcoding. Samples were amplified and given an 8 bp barcode via 11 cycles of PCR amplification using the NEBNext High-Fidelity 2x PCR master mix. Following amplification, fragments were size-selected using Mag-Bind^®^ RxnPure Plus beads. The sample pool was sequenced on a NovaSeq S4 machine at the Harvard Bauer Core to generate ≥40 million reads per sample.

FastQC version 11.9 was used to determine the quality of fastq read files. Because some samples had been run on multiple lanes to achieve the desired number of reads, the reads for each sample were concatenated into a single file for R1 and a second file for R2. NGmerge version 0.3 was used to trim adapters from all reads^52^. Reads were then aligned to the Illumina prebuilt hg38 human reference genome using Bowtie2 (version 2.3.4.1)^53,54^. Reads were then indexed using *samtools index*^55^ and duplicates removed using *picard MarkDuplicates* (version 2.9.0). The resulting files were indexed as before and mitochondrial reads were filtered out (https://github.com/harvardinformatics/ATAC-seq/blob/master/atacseq/removeChrom.py). The resulting .bam files were then used for peak calling via *MACS* software (version 2.1.1.2), using BAMPE and the following flags: --*nolambda –bdg –verbose*^56^. Reproducible peaks across replicates were identified at an IDR threshold of < 0.05, as defined by the IDR statistical test (version 2.0.3). Finally, IDR-called peak sets for autopod skeletal elements were filtered by peaks previously identified in E54 human brain to remove regulatory regions likely associated with general cellular housekeeping processes^30^. Peak sets were merged, subtracted, overlapped, etc. using the appropriate *bedtools* (v2.27.1) functions^57^.

### Human RNA-seq data collection and processing.

Human samples as described above were dissected in 10% FBS/DMEM and a minimum of *n* = 6 per tissue per timepoint was collected. Each element was stripped of soft tissue and collected in a 2-ml tube containing 200 μl of TRIzol and one 5-mm stainless steel bead. Each sample was then homogenized at 50 Hz for 2 min, followed by 1 min on ice, and a second homogenization at 50 Hz for 2 min. Samples were stored at −80°C until RNA extraction was performed. For each RNA extraction, samples were incubated at room temperature for 5 min and then centrifuged at 4°C for 5 minutes at 12,000 × g. The supernatant was collected and transferred to a clean tube, to which 200 μl of chloroform was added per 1 ml of TRIzol. Each sample was then vortexed briefly and incubated at room temperature for 2 minutes before being transferred to a MaXtract tube and centrifuged at 4°C for 5 minutes at 12,000 × g. Following centrifugation, the aqueous phase was removed and transferred to a new microcentrifuge Eppendorf tube and an equal volume of 100% ethanol was added. After completion of this phenol-chloroform step, the RNA extraction continued using the Direct-zol RNA MicroPrep kit following the manufacturer’s protocols and eluted in 15 μl of nuclease-free water. Samples were stored at −80°C until library preparation. To maximize the number of tissues useable from each biological replicate, samples with RNA integrity number (RIN) scores higher than 6 were used for subsequent steps so long as the average RIN for all tissues from that sample was greater than 7.

For cDNA library preparation and sequencing, samples were normalized to a single concentration and libraries were prepared using the Kapa mRNA HyperPrep kit with an input volume of 25 ng per sample following the manufacturer’s protocols. After an initial MiSeq Nano run to assess library quality, samples were repooled as necessary and then sequenced on an Illumina NovaSeq S4 six times to generate ~ 20 million paired-end reads per sample. See SI Appendix, Table S2 for detailed sequencing information for each sample, including index primer, read count, and other information. Six biological replicates per tissue per timepoint were sequenced.

FastQC version 11.9 was used to assess the quality of each fastq read file^58^. Reads for each sample from multiples lanes were concatenated into a single file for R1 and a second file for R2. NGmerge version 0.3 was used to trim adapters from all reads^52^. Next, reads mapping to ribosomal RNA were removed using RiboDetector^59^ version 0.2.7. STAR^60^ version 2.7.1 was used to map reads to the human genome (hg38) with ≥80% of reads uniquely mapping for each sample. RSEM^61^ version 1.3.3 was then used to generate read counts. For all samples, > 90% of reads were mapped uniquely to a gene.

### Mouse ATAC-seq data collection and processing.

All mouse work was covered under the Capellini lab IACUC protocol (#13-04-161-3). E15.5 mouse embryos were collected from pregnant FVB/NJ females and transferred to cold 1x PBS. The fore- and hind limbs of three to five embryos were microdissected under a light microscope in 5% FBS/DMEM on ice. The pooled phalanges (proximal and distal for digit I, proximal, intermediate, and distal for digits III and V) and metapodials were collected separately for digits I, III, and V for both the forelimb and the hind limb. The right and left sides of each element were pooled together, resulting in a total pool of elements from six to ten individual limbs. All embryos in each biological replicate were from the same litter. Collagenase digestion, cell lysis, the transposase reaction, barcoding, and sequencing were all performed as described above for human samples. Data processing was also the same except that reads were aligned to the prebuild Illumina UCSC mm10 genome, E15.5 mouse brain accessibility data was used for the brain-filtering step^62^, and a different version of MACS (3.0.3) was used. See Supplementary Table 5 for detailed sequencing information for each sample, including index primer, read count, and other information.

### Mouse RNA-seq data collection and processing.

E15.5 mouse embryos were collected from pregnant FVB/NJ females and transferred to cold 1x phosphate-buffer saline (PBS). Embryos were microdissected under a light microscope in 10% FBS/DMEM on ice and anatomically sexed^63^. The pooled phalanges (proximal and distal for digit I; proximal, intermediate, and distal for digits III and V) and metapodials were collected separately for digits I, III, and V for both the fore- and hind limbs. The right and left sides of each element were pooled together. All other elements of the RNA collection and extraction protocol were the same as described for human above.

For cDNA library preparation and sequencing, samples were normalized to a single concentration and libraries were prepared using the Takara SMART-Seq v4 Ultra Low Input RNA Kit following the manufacturer’s protocols. Two samples with high concentrations were also prepared using the Kapa mRNA HyperPrep kit with an input volume of 25 ng per sample as per the human samples to detect any effects of library preparation method. After initial pooling, the relative concentrations of each sample were evaluated by running the pool on a MiSeq Nano and the pool concentrated adjusted accordingly. The final pool was then sequenced four times on an Illumina NovaSeq S4 to generate ≥40 million paired-end reads per sample. On average, samples were sequenced at 86 million reads per sample, within the recommended Encyclopedia of DNA Elements (ENCODE) guidelines (www.encodeproject.org/about/experiment-guidelines/). See Supplementary Table 4 for detailed sequencing information for each sample, including index primer, read count, and other information. Six biological replicates of each tissue were sequenced.

### Differential expression and cluster analysis of RNA-seq.

The large number of tissues investigated in this study (20 tissues at two timepoints in human, 12 in mouse) allows for many potentially pair-wise comparisons, however, only a subset of these comparisons is expected to be biologically meaningful. To minimize the number of factors that could cause gene expression differences, samples from a given tissue were only compared with directly adjacent tissues and with the homologous tissue in the other limb type. For humans, adjacent tissues came from sequential digits while in mouse, only digits I, III, V were collected so the nearest digit was used in place of the adjacent digit. Each human tissue was also compared across timepoints. This comparison scheme aims to target only a single variable for any given differential gene calculation, either spatial location within the autopod, limb type, or timepoint. To give an example, the gene expression profile of early samples of hand phalanges from digit III were compared separately with (1) the early hand phalanges of digit II, (2) the early hand phalanges of digit IV, (3) the early metacarpal of digit III, (4) the early foot phalanges of digit III, and (5) the late hand phalanges of digit III.

All differentially expressed genes (DEGs) were identified using DESeq2^64^ version 1.36.0 with a design including the sample type and the replicate ID except for comparisons between timepoints where only timepoint was included in the design. In addition, to identify genes expressed in a gradient across the digits, DESeq2 was rerun at both time points using a likelihood ratio test to compare a model of ~ replicate + digit with a baseline model of ~ replicate. Thresholds for calling DEGs were P_adj_ < 0.05 and |fold-change| >1.5. This approach was used for both human and mouse.

Many of the DEGs identified above are expected to be DEGs in numerous comparisons, e.g., a gene highly expressed in a single tissue will be identified as a DEG in all comparisons involving that tissue. Similarly, many genes may be expressed in specific spatial domains encompassing multiple tissues and cannot be easily identified using a pair-wise approach. Therefore, to identify large patterns of gene expression present in our dataset, we performed a weighted gene co-expression network analysis (WGCNA) to identify clusters of genes with similar expression patterns^65^. The expression of DEGs was normalized using the variance transformation function *vst* in DESeq2. For comparison within a single timepoint, variation associated with biological replicates was removed using *limma:removeBatchEffect)*^66^. This was not used when comparing both human timepoints because this correction also removed timepoint differences that are of biological interest. The *pickSoftThreshold* function was used to estimate the lowest scale-free threshold for each dataset, which resulted in a minimum scale-free topology of 0.9. These soft power thresholds were used to construct signed correlation networks using the *blockwiseModules* function.

Mouse data was processed following the same pipeline except that nearest digits were compared instead of neighboring digits (so digit III was compared to digits I and V instead of II and IV) due to reduced tissue sampling. DEG-calling was repeated for human with the reduced tissue set (digits I, III, V only) at each timepoint to facilitate direct comparison with mouse.

### Comparison of human and mouse ATAC/RNA-seq.

Mouse orthologs of human genes (GRCh38.p14) were downloaded from the Ensembl BioMart database for comparison of gene sets between the two species. For comparison of orthologous regulatory elements, mouse regions (mm10) were lifted over to human genome coordinates (hg38), allowing for multiple matches and a minMatch = 0.1 threshold.

### Identification of accessibility sharing with other elements of the developing skeleton

Regulatory elements accessible in the autopod were intersected with elements accessibility in at least one other developmental skeletal tissue for which data has been generated using the same ATAC-seq protocol^28–30^. This included the proximal and distal ends of the femur, tibia, humerus, and radius, three regions of the scapula (head and neck, blade, acromion), four regions of the pelvis (ilium, ischium, pubis, acetabulum), as well as the thoracic and the lumbar vertebrae. Data were available at both stages for all tissues except the vertebrae, for which only later stage was available.

### Identification of regulatory overlaps with genomic features

To identify potential regulatory elements that were the targets of past positive selection in the human lineage, regulatory element sets were overlapped with human accelerated regions (HARs), which are non-coding regions of the genome marked by accelerated substitution rates in the human lineage compared to other vertebrates^31–38^, human conserved element deletions (hCONDELs)^39,40^, and human ancestor quickly evolved regions (HAQERs), the fasted evolving regions of the human genome^41^. Regulatory element sets were also overlapped with structural divergent regions of the human genome compared to other apes, including large chromosomal inversions and structural divergent regions (SDRs)^41^. As the HAQERs, inversions, and SDR sets were identified using the T2T-CHM13v2.0 genome, these sets were lifted over to hg38 using the liftover file available at https://github.com/marbl/CHM13. Regions flanking inversions and SDRs were obtained for either 500kb or 1Mb windows on either side of these features.

To investigate patterns of intraspecific variation in regulatory elements, the VCF file containing the positions of human SNPs in the dbSNP151 database were downloaded from the NIH website (https://ftp.ncbi.nlm.nih.gov/snp/organisms/) and converted to bed format using the BEDOPS (v2.4.40) convert2bed function^67^. Chimp and gorilla SNP VCF files were downloaded from the Great Apes Genome Diversity Project^68^. We curated common (i.e., minor allele frequency > 0.05) single nucleotide polymorphisms (SNP) from each species. All intersections were performed using *bedtools intersect*^57^.

To compare the number of regulatory elements from distinct sets falling near expressed genes, the coordinates of genes with a minimum normalized expression of 10 in at least one autopod tissue based on the UCSC hg38 refGene list were extended by 500kb in either direction and overlapped with the regulatory element sets using the *bedtools intersect -c* function. Statistical significance was assessed using a two-sided Pearson’s correlation test.

### Gene ontology enrichment analysis

Gene ontology (GO) analysis of gene lists was performed using clusterProfiler with a background set of all genes expressed in at least one of the sampled tissues^69^. Enrichment analysis of genomic region sets was performed using rGREAT (v2.6.0) for five ontologies (“GO Molecular Function”, “GO Biological Process”, “GO Cellular Component”, “Human Phenotype”, “Mouse Phenotype”) using the standard thresholds used in the online version of tool (binomial and hypergeometric P_adj_ < 0.05, binomial fold enrichment ≥ 2)^70,71^.

### Transcription factor (TF) binding motif enrichment analysis

TF binding motif enrichments were calculated in R using MonaLisa^72^ with human PWM matrices from the JASPAR database^73^. Statistical significance was assessed using a negative log10 cut-off of 4. Regulatory element sizes were not standardized for these analyses.

### Calculation of human chimp sequence divergence

Prior to performing this analysis, all elements in the genomic region sets were standardized to a length of 500 bps. To calculate the minimum number of single base pair differences between the human and chimpanzee genomes in each set of genomic regions, we first filtered out any positions that are variable within human, chimpanzees, or gorillas since interspecific differences in autopod morphology are much larger than intraspecific variation and therefore current allelic variants are insufficient to explain the evolutionary changes. VCF lists of variants for each species were obtained as described above and SNPs overlapping autopod regions were extracted using tabix (version 1.13)^74^ and converted to bed format using bedtools and then lifted over to hg38 (for chimp and gorilla). Genomic regions sets were filtered by ENCODE blacklist regions^75^ as well as the SNP sets from each species using bedtools to prioritize high quality genomic sequence without interspecific variants. The resulting regions were then lifted over to the chimpanzee (panTro6) and gorilla (gorGor6) genomes, and the DNA sequence extracted using the appropriate BSgenome package^76^. Regions had to be mappable between species along the entire length of the sequence and have at least 25% sequence identity between either target species (human or chimp) and its outgroups. This threshold was chosen to prioritize comparison of orthologous base pairs rather than faulty alignments or indel-related mismatches because 25% is the expected match for random sequences of the same length. A nucleotide was considered derived in a target lineage if it differed from the nucleotide observed (and likely conserved) in the other two species. This analysis was performed with both human and chimpanzee as the target species. The Zoonomia PhyloP scores for each genomic position were obtained for the human and chimpanzee sets^77^ and compared using two-sided Wilcoxon rank-sum test.

### Segmentation of the adult autopod scans

Raw TIFFs and mesh files for corresponding human and chimp autopods were downloaded from MorphoSource and imported into Amira v.2022.1. Slices showing ossification were segmented, and the selected voxels were assigned to a separate material. To visualize the autopod in 3D and generate a model, the “Generate Surface” and “Surface View” options were used in Amira. The final rendered models were exported as object files (.obj) and visualized using Blender 4.4.

## Supplementary Material

Supplementary Files

This is a list of supplementary files associated with this preprint. Click to download.

- OkamotoAutopodS1HominoidMeasurements.xlsx
- OkamotoAutopodS2HumanRNA.xlsx
- OkamotoAutopodS3HumanATAC.xlsx
- OkamotoAutopodS4MouseRNA.xlsx
- OkamotoAutopodS5MouseATAC.xlsx
- AutopodEvolutionSupplement7132025.docx

## Figures and Tables

**Table 1:** Summary of regulatory element overlaps with genomic features

Genomic Feature	All brain-filtered	Autopod-specific	Autopod-specific, human-mouse conserved	Tissue and timepoint specific
HARs	474	75	27	44
hCONDELs	886	157	59	93
HAQERs	215	35	9	17
Inversions	2511	488	116	365
SDRs	297	61	0	52

HARs, human accelerated regions, hCONDELs, human conserved element deletions, HAQERs, human ancestor quickly evolved regions, and SDRs, structurally divergent regions between humans and other apes.

## Data Availability

All data needed to evaluate the conclusions in the paper are present in the paper and/or the SI Appendix. In addition, all ATAC-seq data raw sequencing fastq files and processed peak bed files and RNA-seq data raw sequencing fastq files and processed read count files have been deposited on National Center for Biotechnology Information Gene Expression Omnibus (GSE281502, GSE283854, GSE284187, GSE286924). The code used to perform these analyses and to generate these figures is available at: https://github.com/aokamoto-bio/Human_Autopod_Evolution.
